# Prevention of activated brown adipose tissue on ^18^F-FDG-PET scans of young lymphoma patients: results of an ancillary study within the EuroNet-PHL-C2 trial

**DOI:** 10.1038/s41598-023-48871-2

**Published:** 2023-12-11

**Authors:** C. Pötzsch, Lars Kurch, S. Naumann, T. W. Georgi, O. Sabri, D. Stoevesandt, M. Cepelova, D. Körholz, C. Mauz-Körholz, D. Hasenclever, R. Kluge

**Affiliations:** 1 Department of Nuclear Medicine, University Hospital of Leipzig, University of Leipzig, Liebigstraße 18, 04103 Leipzig, Germany; 2 Department of Radiology, Medical Faculty of the Martin-Luther-University, Halle (Saale), Germany; 3 Department of Pediatric Hematology and Oncology, University Hospital Motol and Second Medical Faculty of Charles University, Prague, Czech Republic; 4 Department of Pediatric Hematology and Oncology, Justus-Liebig University Giessen, Giessen, Germany; 5 Institute for Medical Informatics, Statistics and Epidemiology (IMISE), University of Leipzig, Leipzig, Germany

**Keywords:** Cancer imaging, Paediatric cancer

## Abstract

Activated brown fat (aBAT) is known to affect the evaluation of ^18^F-FDG PET scans, especially in young patients. The aim of this study was to determine factors influencing the occurrence of aBAT, and to investigate the effectiveness of the two preventive measures, warming and beta-blocker (propranolol) administration. Five-hundred-twenty-eight ^18^F-FDG-PET scans of 241 EuroNet-PHL-C2 trial patients from 41 nuclear medicine departments in Germany and Czech Republic were screened for aBAT. The occurrence of aBAT was analyzed with patient characteristics (age, sex, body mass index, predisposition to aBAT), weather data at the day of ^18^F-FDG PET scanning as well as the preventive measures taken. Potentially important factors from univariate analyses were included into a logistic regression model. Warming as a preventive measure was used in 243 ^18^F-FDG-PET scans, propranolol was administered in 36, warming and propranolol were combined in 84, and no preventive measures were taken in 165 scans. Whereas age, sex and body mass index had no clear impact, there was an individual predisposition to aBAT. Logistic regression model revealed that the frequency of aBAT mainly depends on the outside temperature (p = 0.005) and can be effectively reduced by warming (p = 0.004), the administration of unselective beta-blocker or the combination of both. Warming is a simple, cheap and non-invasive method to reduce the frequency of aBAT. However, the effect of warming decreases with increasing outside temperatures. Administration of propranolol seems to be equally effective and provides advantages whenever the positive effect of warming is compromised. The combination of both preventive measures could have an additive effect.

## Introduction

Activated brown adipose tissue (aBAT) on ^18^F-FDG-PET scans is a well-known problem in children and young adults, which may markedly hamper appropriate scan evaluation^1,2^. This type of fat is rich in mitochondria and has the ability to produce heat, in particular needed by babies and toddlers, to prevent their body from hypothermia. It is frequently located in the neck and the axillae, but also around mediastinal blood vessels, paravertebral ganglia, and organs such as liver, spleen or kidneys^3,4^. Various potential factors influencing the occurence of aBAT are described in literature^5–7^. They can be grouped into patient-related factors and environmental factors^5–7^.

In young lymphoma patients ^18^F-FDG-PET scans are acquired at staging to determine lymphoma spread, and at restaging to evaluate response to chemotherapy^8^. At restaging metabolic activity of lymphoma residues is determined to decide on further treatment, in particular, whether radiotherapy is necessary or not^9,10^. However, residual lymphoma lesions embedded in aBAT are extremely difficult, if not impossible, to evaluate^8^. If an ^18^F-FDG-PET scan is not evaluable, it has to be either repeated, meaning additional radiation burden to the patient or radiotherapy cannot be omitted^8^. However, radiotherapy is highly problematic in the treatment setting of children and young adults with Hodgkin lymphoma because it markedly increases the risk of treatment related late effects such as secondary neoplasms or cardio-vascular diseases^9–14^.

In the second prospective trial of the European Network for Pediatric Hodgkin Lymphoma (EuroNet-PHL-C2; EudraCT: 2012-004053-88; ClinicalTrials.gov Identifier: NCT02797717), ^18^F-FDG-PET was compulsory at staging and following two courses of vincristine, etoposide, prednisone, and doxorubicin (OEPA) chemotherapy. In all patients with a negative interim ^18^F-FDG-PET scan (Deauville scores ≤ 3) irradiation was omitted. Patients with intermediate (Ann Arbor stages IE, IIA plus risk factors, IIB, IIE, IIIA) or advanced stages (Ann Arbor stages IIBE, IIIB, IIBE, all IV) who remained ^18^F-FDG-PET positive (Deauville scores > 3) received another ^18^F-FDG-PET scan at the end of their chemotherapy^15^. Final decision on irradiation fields and irradiation doses was based on randomization (standard arm versus experimental arm) as well as on the results of the two (interim- and end-of-treatment) ^18^F-FDG-PET scans. Thus, evaluability of the ^18^F-FDG-PET images was of utmost importance for precise treatment planning. To prevent aBAT, the imaging protocol of the EuroNet-PHL-C2 trial recommended two measures: Warming the patient and, unless there were contraindications or the patient was younger than 10 years, the administration of an unselective betablocker (propranolol; 1 mg/kg, up to 40 mg in total)^15^.

The aim of this study was to investigate retrospectively different factors, which may have an influence on the occurrence of aBAT on ^18^F-FDG-PET scans and to determine the effectiveness of the two preventive measures (warming, propranolol).

## Methods

### Data sources and data acquisition

Every EuroNet-PHL-C2 trial patient received ^18^F-FDG-PET (as PET/CT or PET/MRI) scans at initial staging and following two cycles of OEPA chemotherapy (early response assessment). Intermediate and advanced stage patients with partial metabolic response (Deauville > 3) after two cycles of OEPA had another ^18^F-FDG-PET scan at the end of their consolidation chemotherapy (late response assessment). The study protocol gave only recommendations on how to perform the ^18^F-FDG-PET scans^15^, but did not define mandatory criteria. Thus, imaging data closely reflect the clinical reality of numerous PET centres.

All imaging data (^18^F-FDG-PET/CT or –PET/MR, CT, MR, bone scans) performed within the EuroNet-PHL-C2 trial underwent reference reading. They were stored on a central server for re-evaluation and research purposes^16^.

For the current study, 528 consecutive ^18^F-FDG-PET/CT or –PET/MR scans of 241 pediatric patients were reviewed. The scans were performed between November 3, 2015 and August 30, 2017 at 41 different nuclear medicine departments in Germany and Czech Republic. All 241 patients had one ^18^F-FDG-PET scan for staging. Upon availability their early (n = 232) and late response assessment (n = 55) scans were considered as well.

The scans were checked for aBAT by two experienced technologists (both > 20 years of working experience). Beforehand, both underwent an intensive training conducted by a nuclear medicine physician who served as the reference reader for the EuroNet-PHL-C2 trial. Subject of the training was a set of 30 ^18^F-FDG-PET/CT or –PET/MR scans in which aBAT was present in varying degrees, and at all typical and less typical sites.

In addition to reviewing the 528 ^18^F-FDG-PET scans for the presence of aBAT, patient age, sex, height and weight were retrieved from the EuroNet-PHL-C2 trial database. Body mass index (BMI) was calculated on the basis of patient’s weight and height. However, since BMI is age dependent, we calculated the so-called BMI_sds using age and gender specific percentiles data from German children and adolescents^17^. The BMI_sds is Z-score based on a standard normal distribution.

Mean outside temperature in the respective town for the period of the ^18^F-FDG-PET scan was taken from public weather sources.

All 41 nuclear medicine departments were asked for their general policy to prevent aBAT and to provide detailed information on preventive measures for every ^18^F-FDG-PET scan included into the analysis.

### Ethics declarations

Written informed consent was obtained from all patients and/or their legal guardians before inclusion into the EuroNet-PHL-C2 trial. The trial was performed in accordance with good clinical practice and the Declaration of Helsinki. The Ethics Committee of the Medical Faculty of the University of Leipzig which is registered as Institutional Review Board (IBF) at the Office of Human Research Protections (OHRP) approved the evaluation presented here (498/17-ek).

### Statistics

This is a retrospectively performed observational study. The preventive measures analyzed were not randomized or prescribed, but followed mainly site-specific policies. Therefore, our strategy of analyses was to first look at single factors (gender, age, BMI_sds, outside temperature, predisposition to aBAT) which may have an impact on aBAT using univariate statistical methods for all scans together (Chi^2^-test for cross-tables and logistic regression for the influence of a metric on a binary factor). A logistic regression model then analyzed potentially important factors, with patient as random effect to account for patient disposition. Data were first collected in SPSS Statistics 23. All analyses were carried out using R, version 4.3 (R Core Team, Vienna, Austria).

## Results

### General results

aBAT was detected on 94 of the 528 ^18^F-FDG-PET scans (17.8%). In 165 of 528 ^18^F-FDG-PET scans (31.3%) no preventive measures against aBAT were taken, whereas at least one preventive measure was taken in 363 ^18^F-FDG-PET scans (68.7%). Further details are listed in Table 1. However, warming was not documented in every individual case, in particular not, when warming was a general policy at the respective nuclear medicine department. For further analysis we, thus, assumed that the policy of warming was strictly followed. This appeared justified since there was no significant difference in aBAT rates between “Warming—general policy*” (153 × no aBAT, 27 × aBAT) and “Warming—confirmed**” (55 × no aBAT, 8 × aBAT) (p = 0.811). Considering this assumption, ^18^F-FDG-PET scans were performed after warming the patient in 243 (46.0%), after administration of only propranolol in 36 (6.8%), and after combining warming and propranolol in 84 scans (15.9%).

**Table 1:** Overview of performed or not performed measures to prevent aBAT.

Preventive measure	Frequency	%
None	165	31.3
Warming (general policy*)	180	34.1
Warming (confirmed**)	63	11.9
Propranolol only (confirmed)	36	6.8
Propranolol (confirmed) and Warming (general policy)	54	10.2
Propranolol (confirmed) and Warming (confirmed)	30	5.7
Σ	528	100.0

General policy (*) means that young patients who undergo ^18^F-FDG-PET scans are regularly warmed before radiotracer injection. Confirmed (**) means that warming and/or administration of propranolol prior to radiotracer injection was documented in the patient file.

### Univariate analysis of factors with possible influence on aBAT

#### Gender

Table 2 shows the frequency of aBAT depending on gender. No aBAT was evident in 211 ^18^F-FDG-PET scans from male (83.4%) and 223 ^18^F-FDG-PET scans from female (81.1%). aBAT was detectable in 42 ^18^F-FDG-PET scans from male (16.6%) and 52 ^18^F-FDG-PET scans from female (18.9%). These numbers suggest that gender did not influence the frequency of aBAT (p = 0.563).

**Table 2:** Frequency of aBAT in relation to gender.

Gender	No aBAT	%	aBAT	%	Σ
Male	n = 211	83.4	n = 42	16.6	253
Female	n = 223	81.1	n = 52	18.9	275
Σ	n = 434	82.2	n = 94	17.8	528

#### Age

Patients included in our analysis were between 2 and 18 years old. They were divided into two groups setting a cut-off at 11 years. The intention of this cut-off was to distinguish pre-pubertal from pubertal or post-pubertal patients.

The numbers in Table 3 indicate that aBAT was more frequent in patients > 11 years (19.8% versus 10.5%, p = 0.031). This result, however, did not remain significant on multivariate analysis (data not shown).

**Table 3:** Frequency of aBAT in relation to age.

	< 11 years	%	≥ 11 years	%	Σ
No aBAT	n = 102	89.5	n = 332	80.2	434
aBAT	n = 12	10.5	n = 82	19.8	94
Σ	n = 114		n = 414		528

#### BMI_sds

**Figure 1:**
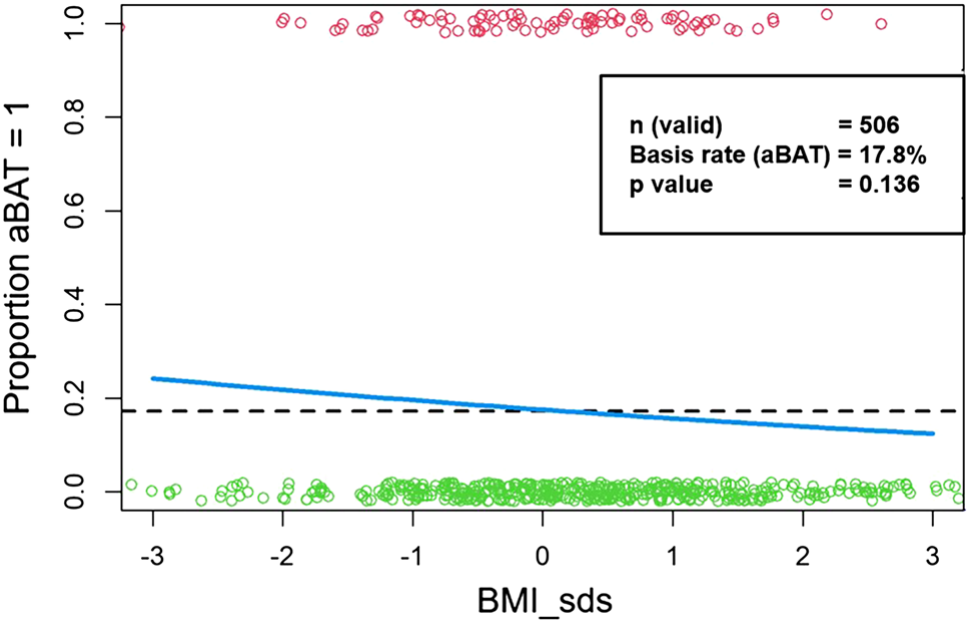
Influence of BMI_sds on the frequency of aBAT (blue curve). Green circles = no aBAT, red circles = aBAT. Dashed line = basis rate of aBAT.

BMI_sds was only calculable for the patients of 506 of the 528 ^18^F-FDG-PET scans as either weight or height were not available. Figure 1 shows the proportion of aBAT (y-axis) as a function of BMI_sds (x-axis). Red circles represent PET scans with aBAT, green circles PET scans without aBAT. The dashed line stands for the baseline rate of aBAT, which is at 17.8%. The light blue logistic regression curve shows a slight decrease in the rate of aBAT from lower BMI_sds (approx. 24%) to higher BMI_sds (approx. 16%). However, this trend was not significant (p = 0.136).

#### Outside temperature

**Figure 2:**
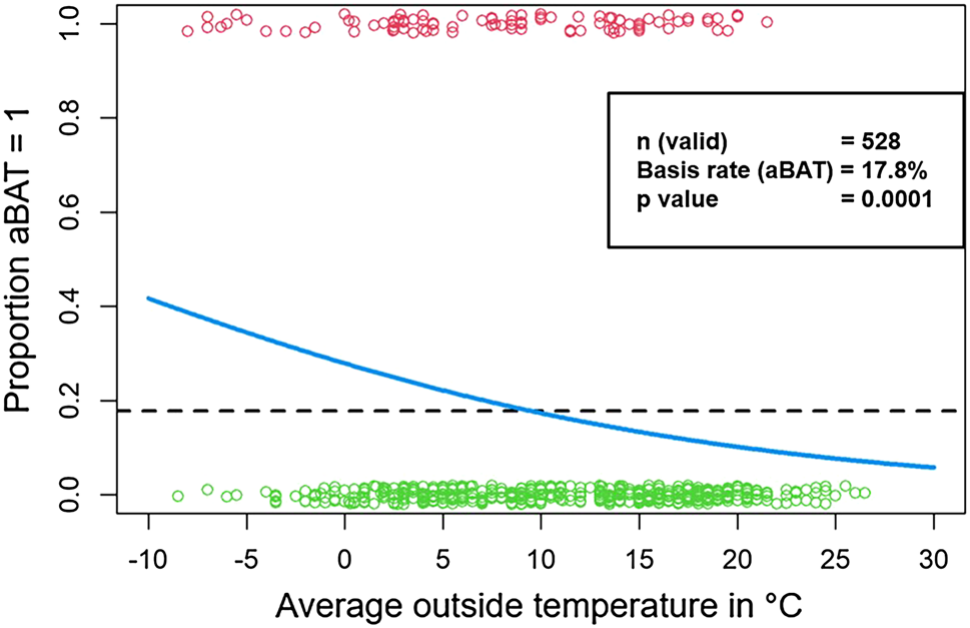
Influence of the average outdoor temperature on the frequency of aBAT (blue curve). Green circles = no aBAT, red circles = aBAT. Dashed line = basis rate of aBAT.

Figure 2 shows the proportion of aBAT (y-axis) as a function of average outside temperature stated in degrees Celcius (x-axis). Red circles represent PET scans with aBAT, green circles PET scans without aBAT. The dashed dark line stands for the baseline rate of aBAT, which is at 17.8%. The light blue logistic regression curve indicates that outside temperature has a clear influence on the occurrence of aBAT. Accordingly, aBAT significantly increased with lower and decreased with higher outside temperatures (p = 0.0001). However, aBAT frequency never reached 0 or 100%, suggesting that the outside temperature was an important, but not the only factor.

### Influence of outside temperature on preventive measures

**Figure 3:**
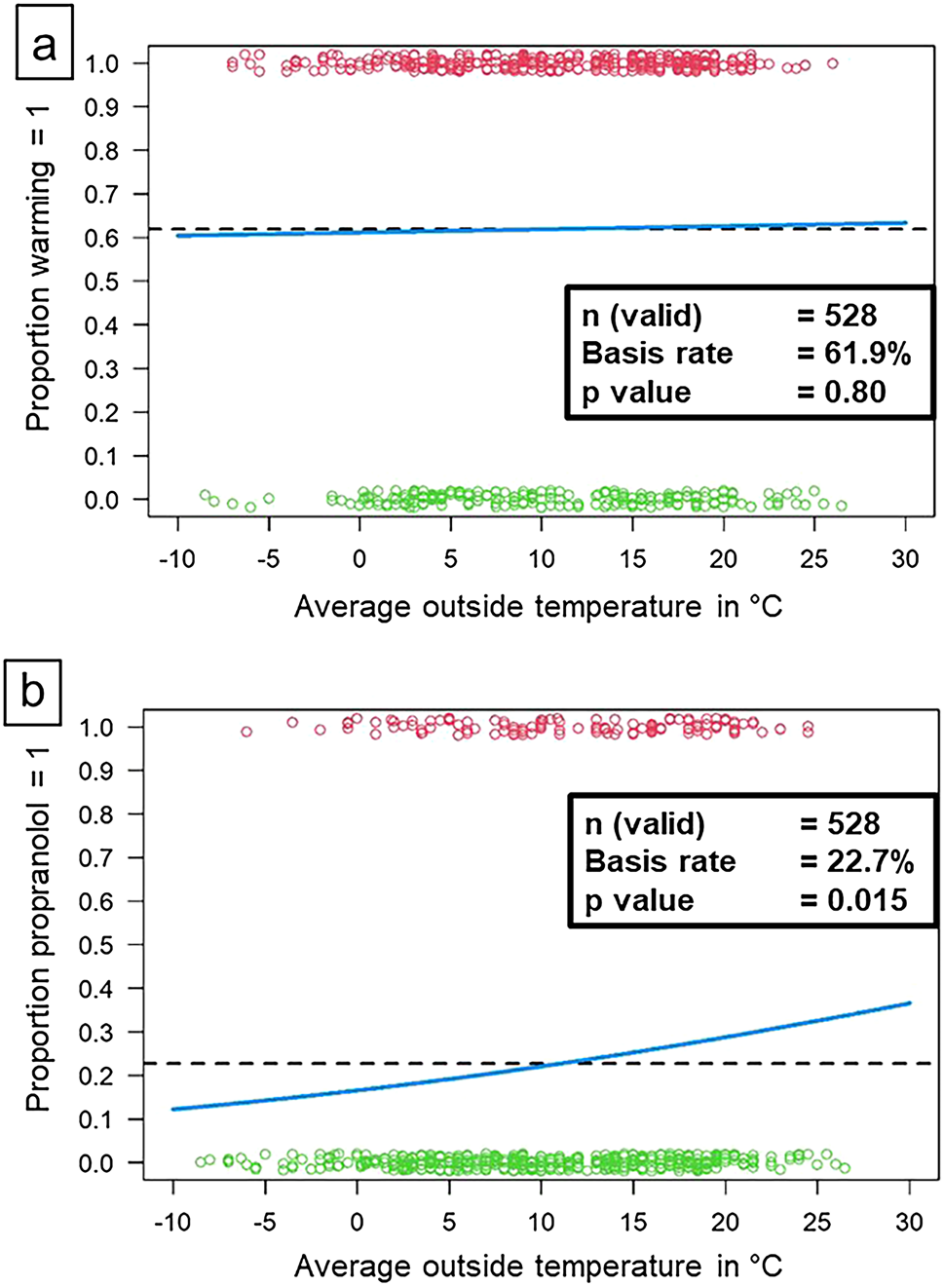
Interrelation between the outside temperature and the execution of preventive measures: (**a**) warming the patient (blue line = proportion of patients warmed), (**b**) administration of propranolol (blue line = proportion of patients in whom propranolol was given). Green circles = no aBAT, red circles = aBAT.

We first explored whether the outside temperature had an influence on the choice of the preventive measure: Fig. 3a displays the proportion of patients who received warming (y-axis) as a function of average outside temperature in degrees Celcius (x-axis). Red circles represent PET scans with aBAT, green circles PET scans without aBAT. The dashed dark line stands for the baseline rate of performed warming as preventive measure, which was at 61.7% (in 327 of 528 PET scans). The course of the light blue logistic regression curve remains flat, indicating that the average outside temperature had no influence on whether the patient was warmed or not (p = 0.80).

As a next step, we conducted the same analysis for propranolol: Fig. 3b shows the proportion of patients who received propranolol (y-axis) as a function of average outside temperature in degrees Celcius (x-axis). Red circles represent PET scans with aBAT, green circles PET scans without aBAT. The dashed dark line stands for the baseline rate of propranolol administration as preventive measure, which was at 22.7% (in 120 pf 528 PET scans). The course of the light blue logistic regression curve increases with increasing outside temperatures, meaning that the use of propranolol increased with higher temperatures. This trend turned out to be significant (p = 0.015).

### Patient predisposition

We looked at pairs of ^18^F-FDG-PET scans of the same patient at staging and response assessment to see whether there is evidence for a patient predisposition for aBAT: 14 of 35 patients (40%) with aBAT and 28 of 196 (14.3%) without aBAT at staging presented with aBAT at response assessment (p < 0.001). This result suggests a patient predisposition.

### Model analysis on the effect of preventive measures

**Figure 4:**
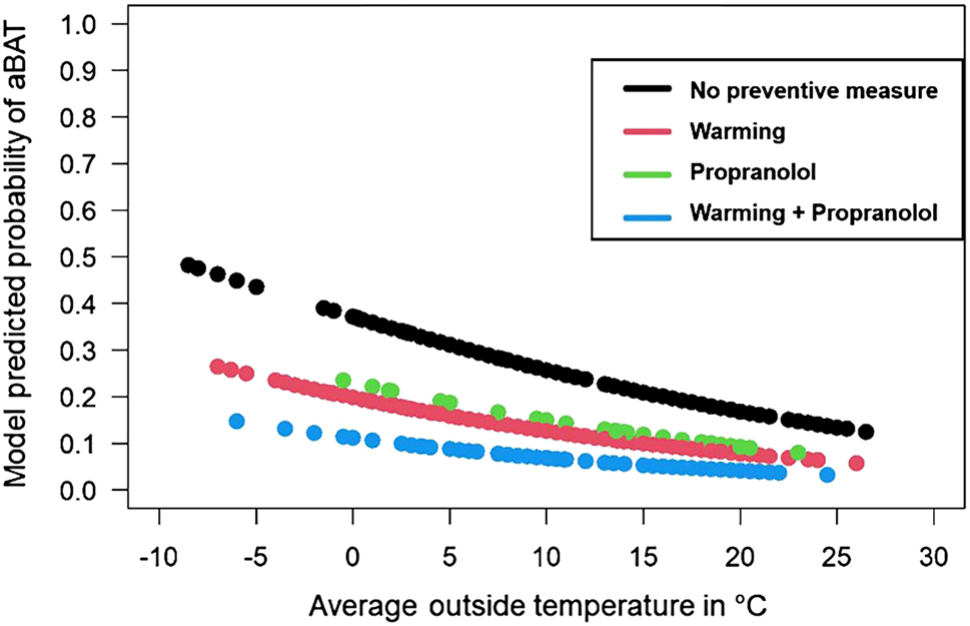
Probability of aBAT as a function of the average outdoor temperature and use of preventive measures.

To synthesize our findings, we fitted a logistic regression model (with patient as random effect to account for multiple images per patient) with all factors found to be relevant in the preceding analyses. From the model, we calculate the predicted probability of aBAT as a function of average outside temperature (in °C) and the preventive measures (warming and/or propranolol or none). The corresponding results are shown in Table 4 and in Fig. 4. Figure 4 displays the probability of aBAT predicted by the model as a function of the average outside temperature (in degrees Celsius) depending on the applied preventive measure (black graph = no preventive measure, n = 165; red graph = warming alone, n = 243; green graph = administering of propranolol only, n = 36; blue = combination of warming and administering of propranolol, n = 84). Table 4 and Fig. 4 demonstrate that both measures were effective to reduce aBAT. While warming patients significantly reduced the occurrence of aBAT (p = 0.004), the administration of propranolol was of borderline significance (p = 0.08) possibly due to the comparatively small number of patients in this group (Table 1). If both preventive measures were combined, there tended to be an additive effect (Fig. 4). Nevertheless, the effect of every preventive measure or its combination was significantly dependent on the outdoor temperature, i.e. the effectiveness increased with lower, but decreased with higher temperatures (Fig. 4).

**Table 4:** Results of the aBAT prediction logistic regression model including average outside temperature and the two preventive interventions (warming, propranolol, and the combination of both).

	Estimate	Standard error	Probability (> │Z│)
(Intercept)	− 0.526	0.281	0.062
Average outside temperature	− 0.054	0.019	0.005
Warming (true)	− 0.87	0.299	0.004
Propranolol (true)	− 0.678	0.387	0.080

## Discussion

aBAT on ^18^F-FDG-PET scans is an issue, especially in young patients with Hodgkin lymphoma. At worst, aBAT impairs the readability of the scans to such an extent that it becomes impossible to determine the degree of metabolic response, which is essential to decide on further treatment. Therefore, the aim of this study was to find out whether preventive measures such as warming and/or propranolol are able to suppress aBAT, and whether patient related and environmental factors influence its frequency. Therefore, we analyzed a total of 528 ^18^F-FDG-PET scans of 241 consecutive patients from the EuroNet-PHL-C2 trial.

Our results show that aBAT frequency did not differ between male and female patients, which is in line with several previously performed studies (one with 385 scans, the other with 2792 scans in pediatric cancer patients)^2,18^. However, there seems to be a difference to adults as a large study with more than 15,000 ^18^F-FDG-PET scans from patients who were on average 61 (± 13) years old, showed that aBAT occurred more frequently in women^6^.

With regard to age, we observed that pediatric patients under the age of 11 had less aBAT compared to patients over the age of 11. This result was of borderline significance on univariate analysis and turned out to be of non-significance on multivariate analysis. Nevertheless, it corresponds well to the trend seen in the data published by Brady et al.: In this study, the frequency of aBAT in patients under the age of 12 ranged from 7 to 17%, whereas it ranged from 22 to 29% in patients in between 12 and 20 years. Gilsanz et al.^19^ came to a similar conclusion. They explained the difference as a matter of pre-pubertal and pubertal status since pubertal status is associated with hormone changes as well as physical adaptations, such as an increase in muscle mass. Growth and sex hormones, in particular, appear to have an influence on aBAT. However, hormonal constellations and interactions are complex which make it extremely difficult to precisely work out its effect on each individual and on the occurrence of aBAT^20,21^. Regarding body composition—determined by BMI_sds—we found no significant association between BMI_sds and the frequency of aBAT even though we observed a slight trend towards higher aBAT rates in patients with lower BMI_sds. The latter trend is backed by research results from an adult population published by Steinberg and colleagues. They were able to demonstrate that aBAT was more frequently in patients with a lower body mass index^6^. Drubach and colleagues came to a similar result in children and adolescents^2^.

We were able to confirm that the occurrence of aBAT is dependent on the outdoor temperature, whereby aBAT frequency increased with decreasing outdoor temperatures and decreased with increasing outdoor temperatures, respectively^6,22,23^. Thus, warming as a preventive measure suggests itself. In our cohort, warming significantly reduced the frequency of aBAT, although the preventive effect of warming diminishes with increasing outdoor temperatures. This result, however, conflicts with findings of a large study including 1290 patients and 2792 scans^18^. The 1290 patients were divided into three groups: The first group (323 patients, 630 scans) received no prevention against aBAT, the second group (345 patients, 705 scans) underwent “warming” by keeping the room temperature constantly at 24 degrees during the ^18^F-FDG uptake phase, and the third group received oral premedication with propranolol 60 min before the ^18^F-FDG injection. The frequency of aBAT on ^18^F-FDG-PET scans was 32.2% in group 1, 40.6% in group 2 and 15% in group 3^18^. These data suggest that warming even increases the rate of aBAT. This is, however, contrary to the experiences of many nuclear medicine physicians, and to the recommendations of current guidelines for PET imaging in children and adolescents^1,24^. A closer look at the “method of warming” used in group 2 provides possible explanations for that discrepancy: three imaging clinics/departments from Cincinnati, Ann Arbor and Memphis participated in this study. Average daily highs are above 24° in Memphis from May to September, in Ann Arbor from June to August, and in Cincinnati from June to September^25–27^. During these periods, 24 degrees room temperature does not mean warming the patient, but rather cooling. Thus, it could well be that the temperature difference between outside and inside triggered aBAT. Another explanation for the discrepant result could be running air-conditioning systems. They create a constant, conditioned airflow inside the room and, thus, also on the skin of the people who are in the room. Thereby, warmth is conduced from the skin surface. With time, this also cools the temperature of the venous blood flowing back to the centre of the body. Cold venous blood, in turn, triggers aBAT^4^.

Another finding of our research was that propranolol as an unselective beta-blocker had the same effect as warming. However, this result was of borderline significance, probably because propranolol was less often used as single preventive measure in our dataset. Nevertheless, this result corresponds well with evidence in the literature. Thereafter propranolol is highly effective in preventing aBAT^1,5,18,28–31^.

Finally, there is the suggestion that combining warming and propranolol may further reduce the risk of aBAT. However, this remains a hypothesis, which requires further confirmation since our data lack the power to prove this additive effect.

Few limitations of our study should be considered: it was a retrospective observational study without randomization. The group in which only propranolol was used was too small to obtain final results on its effectiveness. Information from the centres on the use of air conditioning systems in uptake rooms would have been of interest, since all data came from nuclear medicine departments in Germany and Czech Republic. The use of air conditioning systems is expectedly less frequent in these two countries compared to warmer regions in the European Union (e.g. Spain, Italy) or the United States.

## Conclusion

Warming is a simple, cheap and non-invasive method to reduce the risk of aBAT. However, the effect of warming decreases with increasing outdoor temperatures and might be completely neutralized, e.g. as soon as air conditioning systems are in operation during the tracer uptake phase. The administration of propranolol appears to be similarly effective to warming and may be considered the first choice in countries where air-conditioning is regularly used. Warming and propranolol seem to have additive effects particularly when outdoor temperatures are low.

## Data Availability

The data (tables) that support the findings of this study are available from the corresponding author upon reasonable request.

## Abbreviations

aBAT: Activated brown adipose tissue
BAT: Brown adipose tissue
BMI: Body mass index
EuroNet-PHL-C2: European Network for Pediatric Hodgkin Lymphoma Classical Hodgkin Lymphoma, 2nd generation
^18^F-FDG-PET: ^18^F-Fluorodeoxyglucose positron emission tomography
HL: Hodgkin lymphoma
IBF: Institutional Review Board
OEPA: Vincristine, etoposide, prednisone, doxorubicine
OHRP: Office of Human Research Protections
